# Using *in vivo* transcriptomics and RNA enrichment to identify genes involved in virulence of *Candida glabrata*

**DOI:** 10.1080/21505594.2022.2095716

**Published:** 2022-07-31

**Authors:** Sanne Schrevens, Eric Durandau, Van Du T. Tran, Dominique Sanglard

**Affiliations:** a Institute of Microbiology, University of Lausanne and University Hospital, Lausanne, Switzerland; b Vital-IT Group, SIB Swiss Institute of Bioinformatics, Lausanne, Switzerland

**Keywords:** *Candida glabrata*, urinary tract infection, transcriptomics, bioluminescence, mice, virulence

## Abstract

*Candida* species are the most commonly isolated opportunistic fungal pathogens in humans. *Candida albicans* causes most of the diagnosed infections, closely followed by *Candida glabrata*. *C. albicans* is well studied, and many genes have been shown to be important for infection and colonization of the host. It is however less clear how *C. glabrata* infects the host. With the help of fungal RNA enrichment, we here investigated for the first time the transcriptomic profile of *C. glabrata* during urinary tract infection (UTI) in mice. In the UTI model, bladders and kidneys are major target organs and therefore fungal transcriptomes were addressed in these organs. Our results showed that, next to adhesins and proteases, nitrogen metabolism and regulation play a vital role during *C. glabrata* UTI. Genes involved in nitrogen metabolism were upregulated and among them we show that *DUR1,2* (urea amidolyase) and *GAP1* (amino acid permease) were important for virulence. Furthermore, we confirmed the importance of the glyoxylate cycle in the host and identified *MLS1* (malate synthase) as an important gene necessary for *C. glabrata* virulence. In conclusion, our study shows with the support of *in vivo* transcriptomics how *C. glabrata* adapts to host conditions.

## Introduction

Opportunistic fungal infections, either superficial or invasive, contribute significantly to morbidity and mortality of hospitalized patients [1]. The majority of these infections are caused by *Candida* species, including, principally *Candida albicans* and *Candida glabrata* [2–5]. Interestingly, *C. glabrata* is more closely related to the baker’s yeast *Saccharomyces cerevisiae* compared to *C. albicans* [6–8]. Both *Candida* species developed the ability to infect their host independently, leading to very different infection strategies [5]. For *C. albicans*, several virulence factors have been described. Virulence is considered to be mainly dependent on the ability to switch from yeast to hyphae. This enables the fungus to disseminate through the bloodstream, as yeast, while penetrating epithelial layers, as hyphae. This morphogenic switch is also important for the formation of biofilms, which protect against host defences and antifungal treatment [9]. On top of that, specific gene families responsible for adhesion, proteolytic activity and defence against reactive oxygen species contribute significantly to virulence depending on the infected niche [10,11]. More recently, the metabolic flexibility of *C. albicans* was added to its list of virulence attributes, as it allows the fungus to grow in diverse environments, contributes to lower susceptibility to antifungals and helps in evading host defences [12]. The pathogenesis of *C. albicans* is well studied and more than 300 genes have been associated with reduced virulence in a mammalian host, as well as about 40 genes to avirulence according to the curated data in the candida genome database (Candidagenome.org). For *C. glabrata* on the other hand, much fewer genes have been reported critical for virulence. Some gene families are linked to virulence in *C. glabrata* and among them several cell wall-associated genes. They include proteases called yapsins (*YPS*-genes) and several adhesins with *EPA1* as an important mediator of adhesion [13,14]. *C. glabrata* has also gained the ability to survive in the environment of macrophages [15] and possesses intrinsic high resistance to oxidative stress [16], both of which can contribute to *C. glabrata* persistence in the host.

S.*cerevisiae* and *C. glabrata* underwent a whole genome duplication, which did not occur in *C. albicans*. Furthermore, fewer genes are found in *C. glabrata* compared to *S. cerevisiae*. Gene loss occurred in central carbon metabolism and nicotinic acid biosynthesis [7]. *C. glabrata* is unable to grow on galactose, maltose, sucrose, raffinose or lactose as the sole carbon source, while assimilation of ethanol, glycerol and acetate is strain-dependent [17]. This fungal pathogen can, however, use specific amino acids as carbon source as well as a nitrogen source [18]. Contrary to gene loss, *C. glabrata* gained some genes, specifically in its telomeric regions. These genes encode cell wall proteins with an important function in adhesion to different surfaces [7].

Only three classes of antifungals are regularly used in the clinic, including the fungicidal polyenes and echinocandins and the fungistatic azoles. Polyenes bind directly to ergosterol in the fungal cell membrane, while azoles target the biosynthesis of this sterol. Echinocandins, on the other hand, target the biosynthesis of glucans in the fungal cell wall [19]. *C. glabrata* exhibits intrinsically higher epidemiological cut-off values for azoles compared to other *Candida* spp and rapidly acquires resistance to azoles [20]. Resistance to echinocandins also occurs but is less frequent, while resistance to polyenes is rare [19–21]. Interestingly, *C. glabrata* isolates acquiring rapidly resistance often contain mutations in *MSH2*, a gene of the DNA mismatch repair pathway [22]. The few antifungal targets and the occurrence of drug resistance motivates the need to investigate *C. glabrata* in more detail with regard to its virulence and possible novel targets for therapeutic strategies.

*In vivo* studies with *C. glabrata* are rare and mainly quantified using fungal burden, due to the low morbidity and mortality in mice. This requires euthanasia of the animals followed by organ extraction and homogenization [23–25]. Following developments in *C. albicans*, bioluminescence was recently used to study *C. glabrata* urinary tract infections (UTIs) [26]. This facilitates the tracing of infection in living animals, allowing the use of organs for other applications including transcriptional studies.

Transcriptomics analysis can unveil the pathways that are needed for fungal adaptation inside the host. Host-mimicking conditions have been used for that purpose with *C. glabrata* cells engulfed by macrophages, or in contact with human blood or epithelial cells [27–29]. Transcriptional studies from host tissues face the issue of limited fungal RNA recovery, since only a small percentage of the RNA extracted from infected organs belongs to the fungus. In *C. albicans*, a study was performed using RNA enrichment during the preparation of the sequencing library to increase the recovery of *C. albicans* RNA from total RNA [30]. This transcriptomics study yielded data consistent with other published studies that investigated genes important for virulence [30]. Such an RNA enrichment approach for *C. glabrata* is challenging, since fungal burdens are usually low in infected hosts. Among available animal models for *C. glabrata* infections, urinary tract infections (UTIs) are attractive in this respect, since high fungal burdens can be maintained in infected bladders [26].

UTIs caused by *Candida* species are usually related to indwelling urinary catheters, with fungi causing 2–15% of the infections [31–34]. *C. glabrata* is an important causative agent with 19–48% of UTIs linked to this pathogen, especially when azole antifungals are used [31–38]. In mice, these infections develop in the bladder but rapidly progress into the kidneys and the spleen [26] congruent with kidney infection causing severe symptoms in humans, leading to high mortality [32,35]. Treatment is challenging, especially when the catheter cannot be removed. As such, it is necessary to study the mechanism of pathogenesis of *C. glabrata* and to identify genes important for virulence that could be used as targets for new antifungals in the future.

In this work, we carried out an *in vivo* transcriptomics study, including extensive RNA enrichment for *C. glabrata* RNA. This led to the identification of multiple pathways and four specific genes involved in *C. glabrata* virulence during UTI. We also show that the *C. glabrata* transcriptome diverges from *C. albicans* during infection.

## Materials and methods

### Strains and growth media

The *C. glabrata* luminescent strain SSY21 was pre-grown for UTI infection as described [26]. Deletion and insertion mutants were pre-grown in yeast peptone dextrose (YPD) (2% Bacto Peptone, 1% yeast extract and 2% glucose) at 30°C under continuous shaking. Spot assays were performed on nitrogen starvation (NS) medium (0,17% YNB without amino acids and without ammonium sulphate, 2% glucose, 2% Bacto agar) supplemented with either 0.5% Urea (PlusOne, GE healthcare), 5 mM L-Citrulline (Sigma) or 5 mM L-Tryptophan (Sigma) for *DUR1,2* and *GAP1* mutants and on YNB (0.67% YNB without amino acids, 2% Bacto agar) supplemented with either 2% potassium acetate or 2% ethanol for *MLS1* and *VMA22* mutants. All strains are summarized in Table S1.

### Urinary tract infection in mice using bioluminescence

All animal experiments were carried out according to the approval of the Institutional Animal Use Committee, Affaires Veterinaires du Canton de Vaud, Switzerland (authorization VD1734.5a) at the University Hospital Center of Lausanne. Animals were housed in ventilated cages with ad libidum access to food and water.

The infections were carried out as described in [26]. Briefly, one hour before the infection, 6-week-old female balb/C mice (Charles River, France) were injected with 0.1 mg/kg buprenorphine. Upon anaesthesia with isoflurane (3%), the bladders were emptied and 2.5 x 10^8^*C. glabrata* cells were injected through a catheter (Intramedick,28 mm-61 mm, Clayton medick, Becton Dickinson). On day 3 post infection, 100 microlitre D-Luciferin (18 mg/ml) was injected and bioluminescence was measured in the Bruker in vivo Xtreme II (Bruker BioSpin MRI GmbH, Ettlingen, Germany) while under anaesthesia. Mice were then sacrificed using CO_2_ and the bladder and kidneys were extracted and stored in RNAlater until RNA extraction.

### RNA extraction

For *in vitro* RNA extraction, exponential phase *C. glabrata* cells were centrifuged and the pellet was snap-frozen in liquid nitrogen and further processed similarly to organs. RNA from organs was extracted similarly according to Amorim-Vaz *et al.* [30]. Briefly, organs were removed from RNAlater, snap-frozen in liquid nitrogen and subsequently mashed using a mortar. The resulting powder was resuspended in RNA buffer (0.1 M TrisHCl pH 7.5, 0.1 M LiCl, 10 mM EDTA, 0.5% SDS), TrizolReagent (Ambion) and phenol-chlorophorm-isoamylalcohol (PCI) (Sigma). Acid-washed glass beads (Sigma) were added and mechanical lysis was carried out in the Precellys Evolution instrument (Bertin Technologies). Upon centrifugation, the supernatant was mixed with PCI, vortexed and centrifuged. The resulting supernatant was then mixed with 100% ethanol and further processed with the Directzol RNA MiniPrep kit (Zymo Research) according to instructions. RNA quality was verified using a 5200 Fragment Analyser (Agilent) upon preparation with the high sensitivity RNA kit (Agilent).

### Probe design for RNA enrichment

Non-overlapping head-to-tail 120-nucleotide probes were designed using the eArray software (Agilent Technologies, Santa Clara, CA). Two probe groups were designed. The first one (“set 1”) included a total of 49,789 bait probes to cover 4995 *C. glabrata* ORFs (assembly GCA_002219205.121) lacking cell wall adhesins. The second design (“set 2”) included a total of 49,964 bait probes to cover 5134 *C. glabrata* ORFs that included cell wall adhesins. Gene lists included in the two designs are given in File S1. To avoid cross-hybridization of probes to multiple cell wall adhesin genes, only gene-specific probes were selected. The first 250 nucleotides of each gene were not covered in the bait designs. It was verified that none of the probes could be mapped to the mouse and human cDNA sequences from Ensembl.

### Library preparation, enrichment, and sequencing

RNA libraries for RNA-seq were prepared using the SureSelectXT multiplexed sequencing kit with RNA target enrichment for Illumina (Agilent Technologies) according to the manufacturer’s instructions. The RNA library preparation was essentially similar to a previously published study that established the RNA enrichment but for *C. albicans* RNA [30]. For non-enriched samples of infected organs and *in vitro* grown *C. glabrata*, RNA libraries for RNA-seq were prepared with a TruSeq stranded total RNA library prep kit (Illumina). Before sequencing, libraries were analysed with a fragment analyser automated CE system (Advanced Analytical) to assess quality and fragment size and with a Qubit fluorometer (Invitrogen) to determine cDNA concentration. Libraries were kept at -20°C until sequencing. The resulting libraries were sequenced on an Illumina HiSeq 4000 system at the LGTF (Lausanne Genomic Technologies Facility) from the University of Lausanne.

### Data analysis

Read processing was performed using a homemade bioinformatics pipeline called ANAPURNAseq. A version of the software is available on GitHUB (https://github.com/Navrique/ANAPURNAseq). ANAPURNAseq (Automated Nextflow Alignment Pipeline for Unprocessed RNAseq) performs various read correction steps, aligns corrected reads to one or two reference genomes and finally counts the number of genes that can be associated with a chosen feature (often gene or CDS). The reference genome used in the alignment procedure (the other possible reference genome was not included) was from isolate DSY562 (Bioproject PRJNA374542, assembly GCA_002219205.1). After running ANAPURNAseq, read counts were normalized using the EdgeR package that filtered for i) low read counts and ii) composition bias with TMM (Trimmed means of *M*-value) [39]. Reads were next transformed with the Voom function of the limma package in R [40] and also corrected for batch effect using the SVA package [41]. Differential gene expression (DGE) was performed with a linear model in the limma package [40]. A principal component analysis (PCA) plot of individual samples (Figure S1) showed little variations within replicate samples. Lists of differentially expressed genes can be found in File S2. Raw sequencing reads can be found under Bioproject PRJNA766605.

Gene set enrichment analysis (GSEA) was carried out with a list of genes with *P*-values ≤0.05 and log2 fold-change ≥2 and ≤ -2 (bladder *in vivo* conditions compared to *in vitro*). A file consisting of 167 differential expression datasets (File S4) from published data in *C. glabrata* was used in the GSEA software 4.10, with following analysis parameters: norm, meandiv; scoring_scheme, weighted; set_min, 15; nperm, 1000; set_max, 500. GSEA results were uploaded into Cytoscape 3.0 with the following parameters: *P* value cut-off, 0.01; FDR *q* value, 0.05. KEGG pathway enrichment was performed with the online tool ShinyGO (http://bioinformatics.sdstate.edu/go/) [42] with a filtered *C. glabrata* gene list of up- and down-regulated genes (log2 fold-change ≥2 and ≤ -2; bladder *in vivo* conditions compared to *in vitro* conditions).

The GO term enrichment was obtained with the over-representation analysis on the four gene sets that are commonly down- and up-regulated in *C. albicans* and *C. glabrata* (File S5), using the clusterProfiler package (v4.0.5) [43]. The GO annotation for *C. albicans* SC5314 (genome A22-s07-m01-r126) and *C. glabrata* CBS138 (genome s03-m01-r12) were used. *P*-values were BH-adjusted [44]. The hierarchical clustering was performed on the Wang-measured semantic similarity of GO terms, using the GOSemSim package (v2.18.1) [45]. The plots of GO terms were created with the enrichplot package (v1.12.3) in R v4.1.2.

### qPCR

To validate the RNA-seq data, expression levels of seven genes (*ATH1*, *DUR1,2*, *GAP1*, *MLS1*, *VMA22*, B1J91_K00825 g and B1J91_K02035 g) were measured with qPCR. One µg of RNA extracted from infected bladders or *in vitro* grown cells was converted into cDNA using the PrimeScript™ RT Reagent Kit (Takara) according to manufacturer’s instructions. qPCRs were carried out using 0.2 µM of each primer and 0.2 µM of the TaqMan probe, using the iTaq Universal Probe kit (Biorad) according to manufacturer’s instructions. Sequences of primers and probes are listed in Table S2. Fold change expression was calculated using the ΔΔCt method, amplifying *RDN58* as the housekeeping gene. qPCRs were carried out with a Quantstudio 3 device.

### Construction of Candida glabrata deletion and reintegration mutants

Deletion mutants were constructed with CRISPR/Cas9 approaches, using a fusion PCR product as a repair template. Each repair template was constructed by fusing three different fragments: fragment 1, a homologous region to the promoter of the gene; fragment 2, the nourseothricin resistance marker (*NAT1*) and fragment 3, a homologous region to the terminator of the gene, following a PCR reaction using nested primers. All used primers are listed in Table S3. The repair fragment and the *in vitro* reconstituted RNA-protein complexes (RNPs) were transformed into strain ATCC2001 using electroporation [46]. Gene-specific RNA guides (Table S4) were designed *in silico* using Geneious Prime (Biomatters, Ltd., Auckland, New Zealand). Gene-specific RNA guides were obtained from IDT (Integrated DNA Technologies, Inc., Coralville, IO, USA). Transformants were selected on YPD supplemented with 200 µg/ml nourseothricin. Correct insertions were verified by PCR with primers listed in Table S3.

Reintegrants were constructed using specific plasmid constructs. Each gene was amplified by PCR, including its promoter and terminator, using primers containing restriction sites (SacI and SpeI) (Table S5). PCR products were next cloned into vector pVS29 using SacI and SpeI restriction sites. pVS29 is originating from pVS20 [47] into which the *URA3* gene was replaced by NaeI-SbfI subcloning with the Hygromycin B resistance marker (*HygB*) from pAP599 [48] after PCR amplification with primers HPH-NaeI 5’-CTTAGCCGGCAAGAAATTACCGTCGCTCG-3’ and HPH-SbfI 5’-AATTCCTGCAGGCCCTTTACCTCTATATCG-3’. Plasmids were transformed into each deletion mutant by electroporation and transformants were selected on YPD supplemented with 300 µg/ml hygromycin B.

### Serial dilution assays

Strains were pre-grown overnight in YPD at 30°C under continuous agitation before being 10-fold serially diluted starting from a density with OD_600_ value of 1. Five microlitres of cell suspension were spotted on each plate and plates were incubated at 30°C ranging from 4 to 6 days. Pictures were taken in Alpha Imager 3400 (Alpha Innotech).

## Results

### RNA sequencing of infected bladders and kidneys

**Figure 1.**
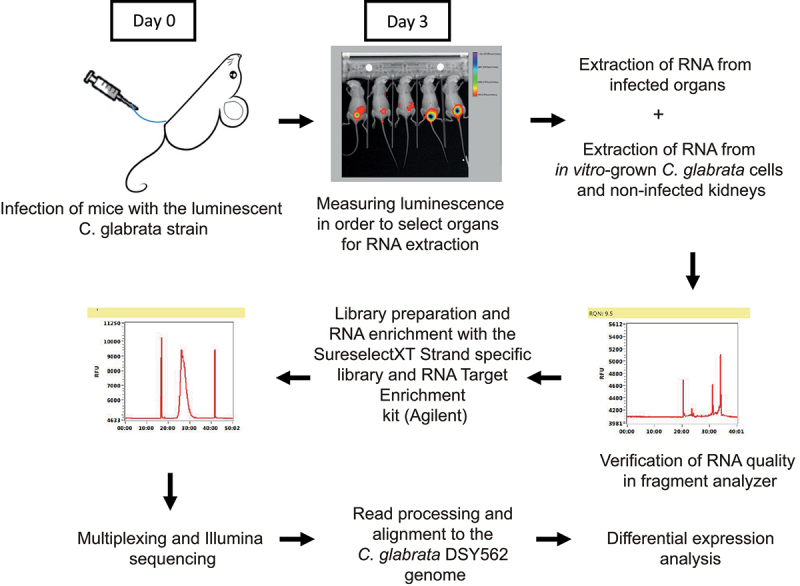
Overview of the RNA sequencing experiment. Stationary phase, luminescent *C. glabrata* cells were injected directly into the bladder of immunocompetent mice via a catheter. At day 3 post infection, bioluminescence was measured and highly infected mice were selected for RNA extraction and processed up to RNA sequencing.

Mice were inoculated with luminescent *C. glabrata* cells (isolate SSY21) directly into the bladder using a catheter. At 3 days post infection, luminescence was measured as described previously [26] to determine which bladders were strongly infected. These bladders, as well as the kidneys from the same animals, were stored in RNAlater immediately after sacrificing the mice until RNA was extracted. RNA was also extracted from non-infected kidneys and from *in vitro* grown luminescent *C. glabrata* cells. Upon RNA quality control, sequencing libraries were prepared and these libraries were subsequently enriched for *C. glabrata* RNA using specific probes, a strategy that we previously applied successfully with mice tissues infected with *C. albicans* [30]. After quality control of the sequencing libraries and Illumina sequencing, reads were processed and aligned to the *C. glabrata* genome (isolate DSY562). These different steps are summarized in Figure 1.

**Figure 2.**
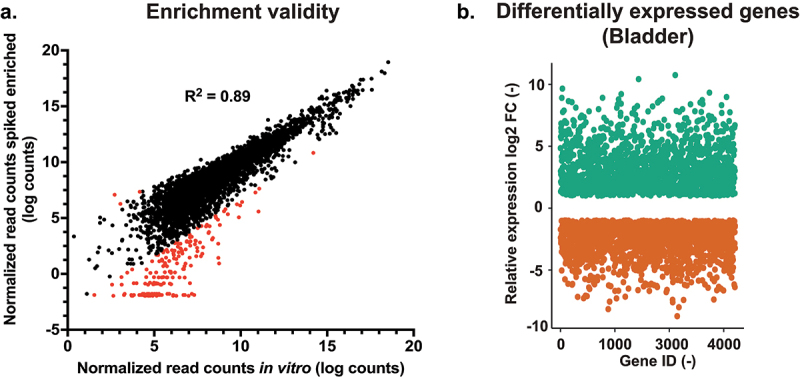
RNA enrichment validity and differential expression between *in* *vivo* and *in* *vitro* conditions. (a) Quality of enrichment was assessed by correlating the normalized read counts of the enriched *in vitro* samples (enriched from a mix with RNA from a non-infected kidney) and the non-enriched *in vitro* samples. Red-labelled data points indicate enrichment bias. (b) Differential expression diagram: more than 1000 genes are up- and downregulated (Log2(fc) ≥1 and ≤1) compared to *in vitro* conditions. FC: fold-change.

File S3 lists all obtained RNA-seq samples, and, if enriched, with which probe set and the amount of total aligned reads for each sample. Enrichment efficiency was calculated based on the percentage of total reads that aligned to the *C. glabrata* genome. Table 1 shows that enrichment of the *in vitro* spiked *C. glabrata* RNA mixed with non-infected kidney RNA and the RNA of infected bladders was very effective. From less than 1% *C. glabrata* RNA in starting material, enrichment yielded more than 60% fungal RNA. The enrichment factor reached values ranging between 750- to 970-fold as compared to non-enriched samples (Table 1). Differences were observed between probe set 2 and probe set 1, the later did not include specific probes for adhesins. Probe set 1 resulted in a slightly more efficient enrichment compared to probe set 2. RNA enrichment from infected kidneys was also effective (about 2000-fold), yet it did not result in high percentages of reads aligning to the *C. glabrata* genome (Table 1). The low percentage of *C. glabrata* reads in the starting material (0.003%) could have been a limiting factor in the enrichment efficiency. Even if our enrichment procedure encountered some limitations, the correlation between normalized read counts of enriched *in vitro* samples (probe set 2) and the non-enriched *in vitro* samples was satisfactory (R^2 = 0.89, Figure 2(a)). While some genes were not enriched due to absence of corresponding probes, a few other genes (3.6% of all probed genes) were not proportionally enriched, which was close to enrichment biases observed with *C. albicans* [30]. More than 1000 genes were significantly upregulated (≥2-fold) in the bladder compared to *in vitro* grown cells and a similar amount was downregulated (≤2-fold). Similar numbers were found for the comparison between infected kidneys and *in vitro* grown cells (File S2).

**Table 1.** Enrichment efficiency for infected organs and spiked samples.

Sample	Reads before enrichment (TruSeq libraries) (%)	Reads after enrichment (Agilent libraries) (%)	Enrichment factor (-)
Infected bladders	0,075 ± 0,01	72,77 ± 0,39 (probe set 1) 56,17 ± 2,77 (probe set 2)	970 (probe set 1) 749 (probe set 2)
Infected kidneys	0,003 ± 0,0003	6,19 ± 1,74 (probe set 1 only)	2063
Non-infected kidneys spiked with *C. glabrata in vitro* RNA	0,29 ± 0,00028	70,15 ± 1,47 (probe set 1) 66,14 ± 1,35 (probe set 2)	242 (probe set 1) 228 (probe set 2)
*C. glabrata in vitro* RNA	88,57 ± 0,63	NA	NA

RNA-seq data were validated by measuring the expression of several selected genes (see below) using qPCR with RNA samples extracted from infected bladders or *in vitro* grown cells. The five genes (*DUR1,2, ATH1, GAP1, MLS1,* and *VMA22)* that were strongly upregulated in the bladder compared to *in vitro* grown cells yielded qPCR results concordant with RNA-seq data (Figure S2). Two genes (B1J91_K00825 g, similar to YGR208w, a *S. cerevisiae* phosphoserine phosphatase; B1J91_K02035 g, similar to YER134c, a putative a *S. cerevisiae* magnesium-dependent phosphatase) significantly downregulated in the bladder compared to *in vitro* conditions by RNA-seq results were also validated by qPCR (Figure S2).

Kidneys and bladder transcriptomes compared to *in vitro* conditions were highly overlapping given that about 84% of genes upregulated in the bladder were also upregulated in the kidneys. Conversely, 77% of genes downregulated in the bladder were also downregulated in the kidneys (Figure S3; see below for additional comments).

### Gene set enrichment analysis shows expected links to other C. glabrata transcriptional studies

**Figure 3.**
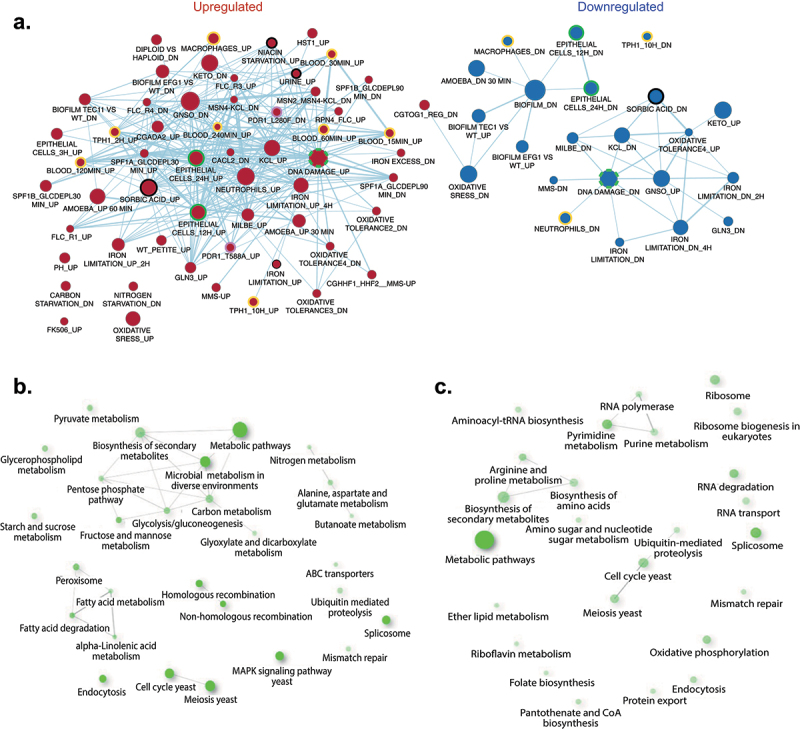
(a) GSEA (Gene set enrichment analysis) of *C. glabrata* genes regulated *in vivo*. The gene list was produced from data in file S4 (“Cg14.Gmt”), in which genes with *P* values of ≤0.05 and log fold-change ≥ 2 (*in vivo* compared to *in vitro*) were chosen. The Cg14.Gmt file contains 167 differential expression data sets from published transcriptional data performed with *C. glabrata*. The list was then imported into the GSEA software 4.10. Analysis parameters were as follows: norm, meandiv; scoring_scheme, weighted; set_min, 15; nperm, 1000; set_max, 500. GSEA results were uploaded into cytoscape 3.0 with the following parameters: *P* value cut-off, 0.01; FDR *q* value, 0.05. Red nodes represent enriched gene lists in upregulated genes from the GSEA. Blue nodes represent enriched gene lists in downregulated genes from the GSEA. Nodes are connected by edges when overlaps exist between nodes. The size of nodes reflects the total number of genes that are connected by edges to neighbouring nodes. Edge thickness reflects the level of confidence between nodes. Labels of nodes indicate specific classes of genes listed in the Cg14.Gmt file. (b) and (c) KEGG pathway enrichment of *C. glabrata* genes up- and down regulated regulated *in vivo*, respectively. The enrichment analysis was performed with the online tool ShinyGO (http://bioinformatics.Sdstate.edu/go/) [42] with a filtered *C.* *glabrata* gene list of up- and down-regulated genes (log2(fc) ≥ 2 compared to in *vitro* growth)

The differential expression in the bladder compared to *in vitro* grown cells was analysed with 167 gene lists of *C. glabrata* regulated genes from several published data sets (File S4). As shown in Figure 3(a) (green circled nodes), our data have several genes in common with those up- and down-regulated in contact with epithelial cells at different time points, especially at 24 h. The convergence of transcriptional profile between these two different conditions suggests that *C. glabrata* cells are in contact with the epithelial cell layers of the bladder. Furthermore, a significant overlap was observed between our data and genes upregulated while grown in urine (black circled node), a significant nutrient source in the bladder, as well as weak acid stress (sorbic acid: black circled node), which probably reflects the low pH that prevails in the bladder [49]. In addition, niacin starvation (black circled node), a condition often linked to the environment of the bladder [50] includes regulated genes with overlap to our data. Data shown in Figure 3(a) also indicate a significant overlap of bladder-dependent transcription with *C. glabrata* profiles obtained with immune cells and blood (orange circled nodes). These results highlight that host-like *in vitro* conditions mirrored the *in vivo* transcriptional landscape of *C. glabrata*. Surprisingly, *PDR1*-dependent expression (violet circled nodes) overlapped with *in vivo* data. Gain-of-function *PDR1* mutations are associated with drug resistance and increased virulence of *C. glabrata* [51], therefore the *in vivo* profile suggests that *C. glabrata* has kept a *PDR1*-like signature to support its lifestyle in the host. We also noticed that a substantial number of regulated genes in the bladder behaved in a similar manner compared to those regulated by DNA damage (green hatched circled node). This suggests a possible link between the response to DNA damaging agents and *C. glabrata* adaptation to *in vivo* conditions.

Taken together, the GSEA revealed that the *in vivo* obtained transcriptional profiles overlapped with other transcriptional studies that included conditions mimicking the host environment.

### KEGG pathway analysis points to the importance of a metabolic shift during in vivo growth

A KEGG pathway analysis was carried out for the differential gene expression in the bladder compared to *in vitro* grown cells. As shown in Figure 3(b,c), the largest difference between the two conditions lies in “metabolic pathways” in both up- and down-regulated genes. Biosynthesis of secondary metabolites, as well as the glyoxylate cycle and fatty acid metabolism seem to be specifically upregulated *in vivo* (Figure 3(a)). In line with the GSEA, where several genes are upregulated in common with the response to DNA damaging agents, pathways for both homologous recombination and non-homologous end joining are upregulated in the bladder. In addition, the signature of MAPK signalling is among the *in vivo* upregulated genes which highlights the need of *C. glabrata* to respond to the unfavourable host conditions. Furthermore, metabolism of specific amino acids (alanine, aspartate and glutamate) seems to be upregulated, while metabolism of other amino acids (arginine and proline) is downregulated. The process of ribosome biogenesis is among down-regulated genes thus probably reflecting that the bladder environment has negative impact on growth of *C. glabrata* compared to *in vitro* conditions.

### In vivo expression of known virulence genes

Proteolytic activity has been linked to virulence both in *C. albicans* and *C. glabrata* [13,52]. *C. glabrata* contains one family of eleven proteases (*YPS* genes), which are glycosylphosphatidylinositol (GPI) anchored cell wall proteins. These proteases are involved in cell wall integrity, adherence to mammalian cells, survival in macrophages and virulence [13]. In our study, we observed that their expression profile differs between the niches of the bladder and the kidneys. While *YPS4, YPS6* and *YPS11* were upregulated in both organs, *YPS1*, *YPS8* and *YPS9* showed bladder-specific upregulation. *YPS2*, *YPS5,* and *YPS10* were only upregulated in the kidneys (Table 2).

**Table 2.** *In vivo* expression of the *YPS* genes.

Gene	Bladder	Fold change Log2 (-)	Kidneys	Fold change Log2 (-)
*YPS1*	Upregulated	1.35	Not regulated	0.88
*YPS2*	Not regulated	0.69	Upregulated	1.04
*YPS3*	Downregulated	-2.04	Not regulated	-0.10
*YPS4*	Upregulated	5.65	Upregulated	6.71
*YPS5*	Not regulated	-0.31	Upregulated	5.2
*YPS6*	Upregulated	3.63	Upregulated	3.74
*YPS7*	Downregulated	-3.2	Downregulated	-2.47
*YPS8*	Upregulated	1.86	Downregulated	-1.09
*YPS9*	Upregulated	3.86	Not regulated	0.99
*YPS10*	Not regulated	0.83	Upregulated	2.10
*YPS11*	Upregulated	4.26	Upregulated	1.53

Another gene family that is important for virulence includes genes encoding adhesins. The most well-known family is the *EPA*-family [14]. However, many other adhesins are present in the *C. glabrata* genome and this varies greatly between strains. The wild-type strain used in our study, DSY562, contains about 101 adhesins or adhesion-like proteins, compared to 63 identified in the reference strain, ATCC2001 [53,54]. However, recent data suggest ATCC2001 may contain up to 83 adhesin-like genes [54]. DSY562 does not contain copies of *EPA3*, *EPA7*, *EPA13,* and *EPA21* [53]. We found that five of the *EPA*-genes were upregulated in the bladder, including *EPA6*, *EPA23*, *EPA9*, *EPA10,* and *EPA20* in addition to four other adhesins (CAGL0I11011g, CAGL0C00253g, CAGL0C01133g, and CAGL0C00896g). Expression patterns of these adhesins are listed in Table 3.

**Table 3.** *In vivo* expression of adhesin genes.

DSY562 locus tag	CBS138 locus tag	Gene name	Fold change Log2 (-)
B1J91_H07469g	CAGL0H07469g	CAGL0H07469g	10.42
B1J91_A01284g	CAGL0A01284g	EPA10	8.90
B1J91_J02508g	CAGL0J02508g	AWP1	8.85
B1J91_L10092g	CAGL0L10092g	NA	7.46
B1J91_C00253g	CAGL0C00253g	CAGL0C00253g	7.10
B1J91_A01366g	CAGL0A01366g	EPA9	6.77
B1J91_J11891g.1	CAGL0J11891g	CAGL0J11891g	4.64
B1J91_J02530g	CAGL0J02530g	CAGL0J02530g	4.19
B1J91_I11011g.1	CAGL0I11011g	CAGL0I11011g	3.69
B1J91_J11176g	CAGL0J11176g	CAGL0J11176g	3.60
B1J91_E00275g	CAGL0E00275g	EPA20	3.53
B1J91_C00110g.1	CAGL0C00110g	EPA6	3.10
B1J91_I10200g	CAGL0I10200g	PWP3	2.96
B1J91_H08844g	CAGL0H08844g	CAGL0H08844g	2.87
B1J91_J00253g	CAGL0J00253g	NA	2.68
B1J91_I00220g	CAGL0I00220g	EPA23	2.43
B1J91_I10246g	CAGL0I10246g	PWP2	1.97
B1J91_L09911g	CAGL0L09911g	CAGL0L09911g	1.12
B1J91_C01133g.1	CAGL0C01133g	CAGL0C01133g	-1.55
B1J91_E02915g	CAGL0E02915g	CAGL0E02915g	-1.59
B1J91_G05896g	CAGL0G05896g	CAGL0G05896g	-1.87
B1J91_C03575g	CAGL0C03575g	CAGL0C03575g	-2.05
B1J91_L06424g	CAGL0L06424g	CAGL0L06424g	-2.54
B1J91_M03773g	CAGL0M03773g	CAGL0M03773g	-3.73
B1J91_D06226g	CAGL0D06226g	CAGL0D06226g	-3.91
B1J91_I10362g	CAGL0I10362g	PWP4	-4.02
B1J91_M14069g	CAGL0M14069g	PWP6	-6.38

Several *C. glabrata* genes have been described as resulting in decreased virulence upon deletion according to the Candida Genome Database including *ATG1, AUS1, HOG1, SKN7, VPH2, VPS15, VPS34, CNA1,* and *CNB1* [55–61]. However, only three of these genes (*AUS1*, *SKN7,* and *CNA1*) were significantly upregulated in our study compared to *in vitro* conditions (File S2).

### Comparison between the bladder and the kidneys transcriptomes

Our data revealed high overlap between the blabber and the kidney transcriptomes (Figure S3). Given that these host environments are quite different, this was unexpected. Besides the niche-specific gene expression as mentioned above, several other genes exhibited inversed regulation patterns that were dependent on the infection site. For example, *WAR1*, a transcription factor involved in the regulation of genes involved in weak acid stress [62] is upregulated in the bladder (log2 FC:1.4), while downregulated in the kidneys (log2 FC:-3.6). These data are consistent with the differences in environmental pH between the bladder and the kidneys [49]. *ATO3*, a putative ammonium transporter, is downregulated in the bladder (log2 FC: -1.6), however strongly upregulated in the kidneys (log2 FC: 4.6). These data reflect that *C. glabrata* grows in the two organs in which ammonium availability is different. It could also link to the necessity of ammonium export to counteract acidification of the phagolysosome upon macrophage phagocytosis, as was shown for *C. albicans* [63].

### Deletion of specific highly differentially expressed genes decreases virulence

In order to address the relevance of highly regulated genes in the process of *C. glabrata* infection in the UTI model, we selected four genes (*DUR1,2, GAP1, MLS1,* and *VMA22*) that were highly upregulated in the bladder compared to *in vitro* conditions. *In vivo* upregulated genes only were chosen, since we hypothetized that they were important for the lifestyle of *C. glabrata* in host tissues. In addition, these genes were selected since they have a known homolog in *S. cerevisiae*, which facilitates *in vitro* phenotypic verifications. *C. glabrata* deletion mutants of each of these genes were tested with serial dilution assays and then used to infect mice. In general, deletion of each of the four selected genes did not affect growth compared to the wild-type parent (Figure S4).

**Figure 4.**
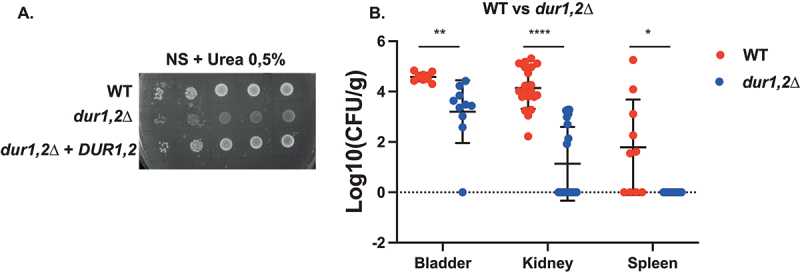
Deletion of *DUR1,2* strongly impairs growth on urea as the sole nitrogen source, as well as virulence in the urinary tract. (a) Serial dilutions of each strain were spotted on nitrogen starvation (NS) medium, containing 0.5% urea as the sole nitrogen source and grown for 4 days at 30°C. (b) Ten mice per group were infected with either the wild type or the mutant strain, by injection (through a catheter) into the bladder. On day 3 post infection, animals were euthanized and bladders, kidneys and spleens were collected for quantification of the fungal burden. (Significance symbols: **** *p* < 0.0001, *** 0.0001 < *p* < 0.0001, ** 0.001 < *p* < 0.01, * 0.01 < *p* < 0.05).

Deletion of the urea amidolyase, *DUR1,2*, resulted in strongly impaired growth on nitrogen starvation (NS) medium containing urea as the sole nitrogen source (Figure 4) [64]. Given that urea is a dominant nitrogen source in urine, *DUR1,2* could be important for colonization of *C. glabrata* of the bladder. Consistent with this hypothesis, *DUR1,2* deletion resulted in significantly reduced virulence in the UTI model. The deletion strain showed reduced colonization of the bladder and the kidneys and seemed unable to colonize the spleen (Figure 4).

**Figure 5.**
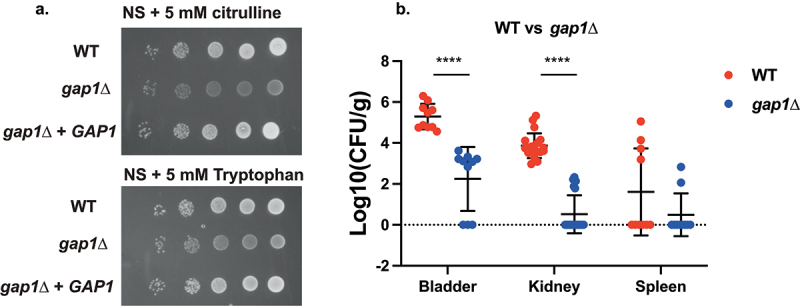
Deletion of *GAP1* strongly impairs growth on citrulline or tryptophan as the sole nitrogen source, as well as virulence in the urinary tract. (a) Serial dilutions of each strain were spotted on nitrogen starvation (NS) medium, containing 5 mM of either L-citrulline or L-tryptophan as the sole nitrogen source and grown for 4 days at 30°C. (b) Ten mice per group were infected with either the wild type or the mutant strain, by injection (through a catheter) into the bladder. On day 3 post infection, animals were euthanized and bladders, kidneys and spleens were collected for quantification of the fungal burden. (Significance symbols: **** *p* < 0.0001, *** 0.0001 < *p* < 0.0001, ** 0.001 < *p* < 0.01, * 0.01 < *p* < 0.05).

The general amino acid permease and receptor, *GAP1*, is well studied in *S. cerevisiae* as a major regulator of nitrogen metabolism in yeast [65–67]. Since several nitrogen-metabolism-related pathways were shown to be differentially regulated *in vivo* according to the KEGG pathway analysis, we hypothesized that amino acid uptake and signalling could be important for virulence. Deletion of *GAP1* in *C. glabrata* resulted in impaired growth on NS medium supplemented with either L-citrulline or L-tryptophan as the sole nitrogen source (Figure 5), which is consistent with *S. cerevisiae* phenotypes [65]. Furthermore, *GAP1* deletion resulted in a strong decrease in colonization of the bladder and kidneys in the UTI model, without abolishing spleen colonization (Figure 5).

**Figure 6.**
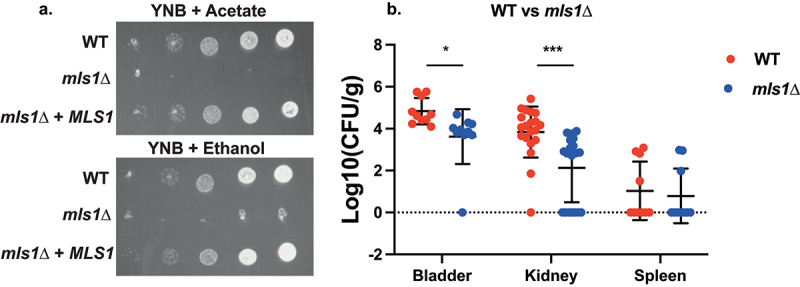
Deletion of *MLS1* abolishes growth on acetate or ethanol as the sole carbon source, as well as virulence in the urinary tract. (a) Serial dilutions of each strain were spotted on minimal medium (YNB), containing 2% of either acetate or ethanol as the sole carbon source and grown for 6 days at 30°C. (b) Ten mice per group were infected with either the wild type or the mutant strain, by injection (through a catheter) into the bladder. On day 3 post infection, animals were euthanized and bladders, kidneys and spleens were collected for quantification of the fungal burden. (Significance symbols: **** *p* < 0.0001, *** 0.0001 < *p* < 0.0001, ** 0.001 < *p* < 0.01, * 0.01 < *p* < 0.05).

The malate synthase gene, *MLS1*, is a part of the glyoxylate cycle that bypasses part of the Krebs cycle in order to retain carbon in limiting environments [68]. Given that the glyoxylate cycle was upregulated in the UTI model according to the KEGG pathway analysis, *MLS1* could be important for virulence in this model. Deletion of *MLS1* in *C. glabrata* resulted in abolishment of growth on minimal medium (YNB) with either acetate or ethanol as a carbon source (Figure 6). This is consistent with *S. cerevisiae* phenotypes; however, in baker’s yeast the defect is also displayed on rich medium (YP) supplemented with acetate or ethanol [68,69], which was not the case for *C. glabrata* (data not shown). The deletion strain was significantly impaired in colonization of the bladder and the kidney, while spleen colonization was not affected (Figure 6).

**Figure 7.**
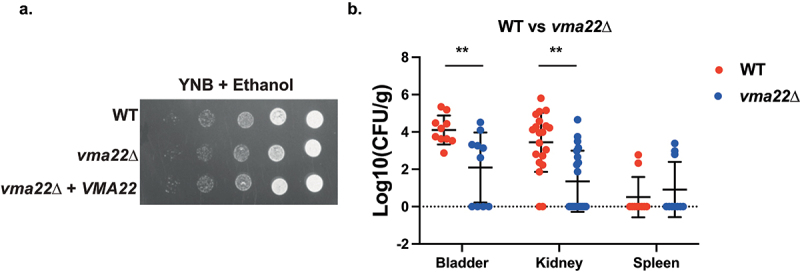
Deletion of *VMA22* results in decreased virulence in the urinary tract, even though no strong growth defects were found *in vitro*. (a) Serial dilutions of each strain were spotted on minimal medium (YNB), containing 2% ethanol as the sole carbon source and grown for 6 days at 30°C. (b) Ten mice per group were infected with either the wild type or the mutant strain, by injection (through a catheter) into the bladder. On day 3 post infection, animals were euthanized and bladders, kidneys and spleens were collected for quantification of the fungal burden. (Significance symbols: **** *p* < 0.0001, *** 0.0001 < *p* < 0.0001, ** 0.001 < *p* < 0.01, * 0.01 < *p* < 0.05).

Vacuolar acidification is important for cellular pH homoeostasis, as well as ion balance and nutrient trafficking throughout the cell and critical for cellular function [70]. *VMA22* is an adaptor that helps assemble the Vacuolar-ATPase, the main actor in vacuolar acidification. Deletion of this gene in *S. cerevisiae* results in strong phenotypes, such as reduced growth in the presence of hydrogen peroxide or inability to metabolize glycerol [71,72]. *VMA22* deletion in *C. glabrata*, however, did not lead to clearly different *in vitro* phenotypes compared to wild type (Figure 7). It did, however, cause a significant decrease in colonization of the bladder (Figure 7).

### Comparison between gene expression during C. albicans and C. glabrata infection

In the work performed in our group by Amorim-Vaz *et al.* [30], *in vivo* transcriptomics was carried out for *C. albicans* during invasive infection in mice by RNA enrichment from infected kidneys. Even though both the model (invasive infection vs UTI) and the timepoint (16 h vs 3 days) are different, it is interesting to look at the most highly significantly expressed genes in their work compared to the *in vivo* expression profiles of *C. glabrata*. Table 4 lists the top 20 most upregulated genes in *C. albicans* during invasive infection and, if existing, their *C. glabrata* counterpart and associated differential expression levels. Twelve of the *C. albicans* genes did not have orthologs in *C. glabrata* and only three orthologs were upregulated during *C. glabrata* UTI. These upregulated genes in common between both studies include a sugar transporter, *HGT12*, a secreted aspartyl protease, *SAP5*, and a transcription factor, *UME6*. Only *UME6* was linked to virulence in *C. albicans* up to now [73]. Transcription factors do not always regulate the same processes in different fungal species (so-called “transcriptional re-wiring”) and as such the *UME6* ortholog may not be necessarily involved in virulence in *C. glabrata*.

**Table 4.** Comparison between most highly significantly upregulated genes in *C. albicans* invasive infection vs *C. glabrata* UTI.

ORF name C. albicans	Gene name C. albicans	Fold change Log2 in kidneys (16 h) (-)	ORF name C. glabrata	Fold change Log2 in bladder (3 days) (-)	Fold change Log2 in kidneys (3 days) (-)
orf19.7455	-	11.80	No ortholog	-	-
orf19.1321	*HWP1*	10.63	No ortholog	-	-
orf19.6028	*HGC1*	10.39	CAGL0I05852g	1.75	2.47
orf19.3374	*ECE1*	9.40	No ortholog	-	
orf19.1363	-	8.53	CAGL0E05456g	1.26	2.0
orf19.5636	*RBT5*	8.43	No ortholog	-	-
orf19.2060	*SOD5*	8.43	No ortholog	-	-
orf19.7094	*HGT12*	8.41	CAGL0J09020g	4.85	3.64
orf19.1816	*ALS3*	8.31	CAGL0G04125g	-3.42	4.32
orf19.5585	*SAP5*	8.25	CAGL0E01793g	3.32	3.74
orf19.1822	*UME6*	8.11	CAGL0F05357g	3.84	2.64
orf19.2457	-	7.91	No ortholog	-	-
orf19.5952	-	7.84	No ortholog	-	-
orf19.2062	*SOD4*	7.71	No ortholog	-	-
orf19.2061	-	7.56	No ortholog	-	-
orf19.4599	*PHO89*	7.21	No ortholog	-	-
orf19.1264	*CFL2*	7.08	CAGL0C03333g	-0.95	-0.72
orf19.1930	*CFL5*	6.96	CAGL0C03333g	-0.95	-0.72
orf19.113	*CIP1*	6.82	No ortholog	-	-
orf19.6148	-	6.69	No ortholog	-	-

When comparing the most highly differentially expressed genes in *C. glabrata* in the kidneys to *C. albicans* genes upregulated in invasive infection (from kidneys), only nine genes have orthologs in *C. albicans* (Table 5). Four of these orthologs were not differentially expressed in *C. albicans* during infection. Only *HST6* and orf19.7588 are upregulated *in vivo* in *C. albicans* as well, although to a much lower extent than in *C. glabrata*. Interestingly, *UPC2*, *GAT1,* and *ATC1* are downregulated during infection in *C. albicans* while being strongly upregulated in *C. glabrata*. *UPC2* and *GAT1* are transcriptional regulators involved in sterol synthesis and nitrogen catabolite repression, respectively. *UPC2* has two orthologs in *C. glabrata*, *UPC2A* (CAGL0C01199g; downregulated *in vivo*) and *UPC2B* (CAGL0F07865g: upregulated *in vivo*), but only *UPC2A* is involved in sterol homoeostasis in this species [74]. Therefore, it is possible that the true *C. glabrata UPC2* ortholog to *C. albicans* is *UPC2A* (CAGL0C01199g) (see also below). We showed here that nitrogen uptake and metabolism are important for *C. glabrata* virulence. It is possible that the *GAT1* homolog is important for this function. Downregulation of this gene in *C. albicans* could mean that nitrogen catabolite repression is less important *in vivo* for this species, or that it is regulated differently. Atc1 is a cell wall-linked trehalase enzyme. *C. glabrata* trehalase enzymes were recently shown to be important for stress resistance [75], which is one of the factors important for the maintenance of fungal virulence.

**Table 5.** Comparison between strongest upregulated genes in *C. glabrata* UTI (kidneys) vs *C. albicans* invasive infection.

ORF name C. glabrata	Fold change Log2 in Kidneys (3 days) (-)	ORF name C. albicans	Gene name C. albicans	Fold change Log2 in kidneys (16 h) (-)
CAGL0I09680g	11.36	CR_10120C_A/orf19.7588	-	1.38
CAGL0F04763g	10.13	No ortholog	-	-
CAGL0F07865g	9.71	C1_08460C_A/orf19.391	*UPC2*	-1.99
CAGL0K00363g	9.67	C3_06510C_A/orf19.7440	*HST6*	2.24
CAGL0J11550g	9.64	No ortholog	-	-
CAGL0L03377g	9.61	C3_04020C_A/orf19.2808	*ZCF16*	Not regulated
CAGL0D03784g	9.46	CR_09720W_A/orf19.6590	*VMA22*	Not regulated
CAGL0K07634g	9.03	C4_05880W_A/orf19.1275	*GAT1*	-1.24
CAGL0A01826g	9.00	No ortholog	-	-
CAGL0D06028g	8.96	C1_00230C_A/orf19.6080	*BFA1*	Not regulated
CAGL0I04048g	8.88	No ortholog	-	-
CAGL0C03784g	8.82	No ortholog	-	-
CaglfMp03	8.76	No ortholog	-	-
CAGL0B04213g	8.71	No ortholog	-	-
CAGL0B03201g	8.60	No ortholog	-	-
CAGL0K05137g	8.54	C1_06940C_A/orf19.6214	*ATC1*	-1.12
CAGL0L09273g	8.47	No ortholog	-	-
CAGL0K06303g	8.34	C2_03180C_A/orf19.5776	*TOM1*	Not regulated
CAGL0B01925g	8.33	No ortholog	-	-
CAGL0I10725g	8.25	No ortholog	-	-

**Figure 8.**
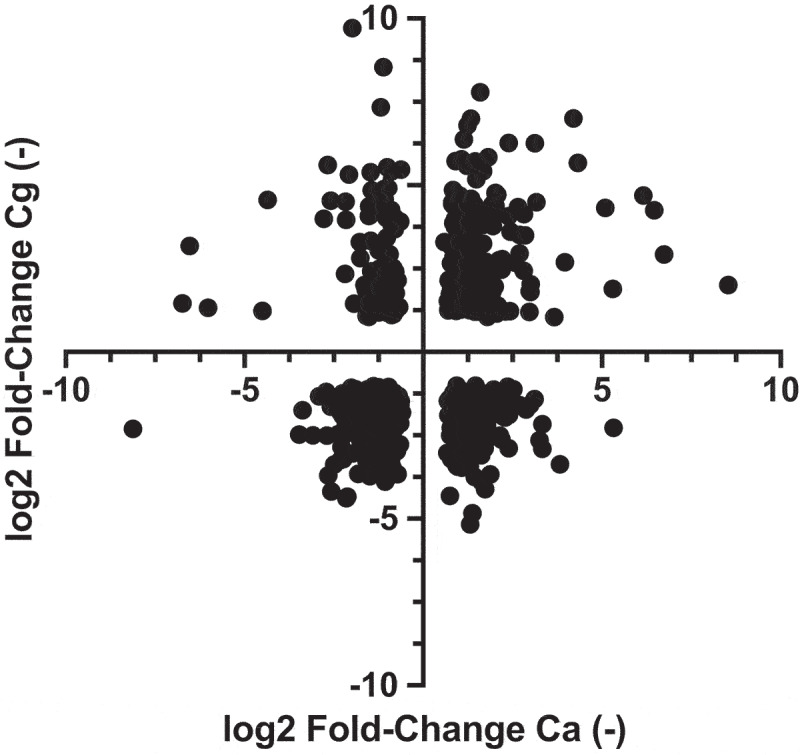
*In vivo* regulation of *C. albicans* and *C. glabrata* orthologs. The orthologs between both species were obtained from the public Candida genome database. The *C. albicans* RNAseq data were obtained from the work of Amorim-Vaz *et al*. [30] taking the 16 h time point of mice infection. The *C. glabrata* RNAseq data were from infected kidneys (File S5). Data were filtered by *p*-value (<0.05).

The divergence between the *C. glabrata* and *C. albicans in vivo* transcriptomes can be best observed when comparing all regulated ortholog genes (593) between the two species (Figure 8). When examining regulated orthologs between the two species, 49% were coregulated, while the remaining were inversely regulated.

The set of commonly regulated genes compared to *in vitro* conditions comprises 120 and 172 up- and downregulated genes between both species recovered from kidneys, respectively (File S5). A GO term enrichment analysis based on gene ontology specific for each species revealed commonly regulated processes. Commonly upregulated genes included biological processes related to response to stress and starvation as well as positive regulation of transcription (Figure S5). This signature reflects that the two fungal species have to cope with an unfavourable host environment. Among the commonly downregulated genes (Figure S6), enriched biological processes participated to translation and ergosterol biosynthesis. Decreased translation revealed by downregulation of several ribosomal genes suggests slow growth of both fungal species in the host. Decreased expression of genes involved in sterol biosynthesis during infection is still difficult to interpret, however is consistent with downregulation of *UPC2A* (a regulator of sterol biosynthesis) in *C. glabrata*, as mentioned above.

## Discussion

In this work, we successfully carried out *in vivo* transcriptomics upon RNA enrichment with the Sureselect RNA enrichment method. Similar to the work of Amorim-Vaz *et al*. performed in our group [30], we managed to quantitatively recover fungal RNA from most fungal genes in total RNA extracted from infected animal organs. Amorim-Vaz *et al*. [30] discovered that only few genes were not proportionally enriched, which was explained by insufficient bait coverage and low GC content of the probes. In addition, we noticed that a limiting factor in the enrichment procedure to high read mapping percentages was the amount of fungal RNA in the starting material. Samples containing 0.0066–0,3% of fungal RNA could be enriched to high percentages, while samples originating from infected kidneys (only 0,003%) did lead to efficient enrichment, but not to high read mapping percentages (Table 1). This is consistent with enrichment efficiencies reported in the previous study [30]. Another alternative to measure *in vivo* gene expression for pathogens is the nanoString nCounter platform. It can, however, only test a limited amount of genes at the same time, making it unable to get a full transcriptome [76,77]. So far, this approach has been used to investigate specific genes during infections of *C. albicans, Aspergillus fumigatus* and *Cryptococcus neoformans* [77–81].

With a probe set including known adhesin genes from isolate DSY562, we showed that several adhesins were strongly upregulated in the bladder compared to *in vitro* growth conditions. Interestingly, the strain DSY562 used in the present study contains significantly more adhesin genes compared to the reference strain ATCC2001 [53]. DSY562 was originally recovered from an AIDS patient with an oral *Candida* infection [82]. Its adhesin repertoire might have been adapted to this host niche. It would be interesting to assess expression of adhesins in this strain during oral infection to determine if a specific adhesion profile exists for each niche.

The Gene Set Enrichment Analysis (GSEA) used in the present study showed that our data overlapped significantly with several other *in vitro* conditions mimicking the host environment, which was also the case for the *C. albicans* study [30]. However, overlap was not only detected with conditions specifically mimicking the *in vivo* environment. Interestingly, many of *in vivo* upregulated genes overlapped with genes upregulated in response to DNA damaging agents [83]. Part of these overlapping genes are homologs to the *S. cerevisiae* mating genes, which were identified by Shor and Perlin [84]. The significance of the DNA damage response in the context of *in vivo* conditions still remains puzzling. The factor that is shared between both this *in vitro* (DNA damage) and the *in vivo* (UTI) situation is stress. Therefore, it is possible that the overlap between these two conditions rather reflects the signature of stress.

In the UTI model, we infected mice directly into the bladder, from where infection spreads towards the kidneys and the spleen [26]. As of yet, it is not clear how the fungus migrates to the kidney and the spleen; it is, however, likely that colonization of the kidneys happened through the ureter. In the invasive infection model, fungal cells are directly injected into the bloodstream, which carries them towards the kidneys and many other organs, where they are either killed or can persist depending on the species [5]. The question remains how the transcriptomics data presented here are relevant in the context of a different infection model, such as invasive infection. Interestingly, *DUR1,2* was shown to be important during invasive infection of *C. albicans* in mice. In the absence of this gene, a more balanced immune response was measured compared to wild type, suggesting that *DUR1,2* contributes to renal failure by unbalancing the host response [85]. In our UTI model experiments, deletion of *DUR1,2* led to a strong reduction in colonization of both the bladder and the kidneys and the inability to colonize the spleen. It remains to be determined whether deletion of *DUR1,2* has an effect on the host immune response or whether its effect on virulence mainly stems from the inability to assimilate urea as a nitrogen source. In *C. albicans DUR1,2* is coregulated with the urea transporter *DUR3* [25], which is not the case in *C. glabrata* taken from our data. It is possible that this transporter is post-translationally regulated in *C. glabrata*. Additionally, Brunke *et al*. [86] showed that *DUR1,2* deletion resulted in reduced virulence in the fruit fly as well as during invasive infection in mice. It is as such possible that the other virulence genes identified in our work are important during invasive infection.

The general amino acid permease, *GAP1*, has been studied extensively in *S. cerevisiae*. It is required for transport of L-citrulline, while it also transports a wide variety of other amino acids. Furthermore, *GAP1* is necessary for the activation of the PKA pathway upon addition of amino acids in the presence of a fermentable carbon source [65,66]. Interestingly, PKA activation and transport are independent [65,66]. Whether the *C. glabrata GAP1* is also a transceptor is not known. Deletion of the gene did strongly affect virulence in the UTI model. Interestingly, *C. albicans* does not have a clear *GAP1* homolog; it has six genes that are more or less homologous to the *S. cerevisiae GAP1*. Each of these homologs has their own amino acid transport specificity, while *GAP4* even transports S-adenosylmethionine (SAM) [87,88]. Signalling activity was not shown for any of the *C. albicans GAP* genes. We identified two genes involved in nitrogen metabolism, uptake and possibly regulation to be involved in virulence, namely *DUR1,2* and *GAP1*. However, other genes involved in nitrogen sensing and response were strongly upregulated in the bladder. One of them is the *C. glabrata* homolog of *S. cerevisiae SSY1* and *C. albicans CSY1*, CAGL0E01089g. *SSY1* is a part of the SPS amino acid sensing system, which senses amino acids extracellularly and then activates amino acid catabolism [89]. *C. albicans CSY1* was shown to be required for amino-acid-induced morphogenesis [90], a process that has been linked to virulence in this organism [91]. Furthermore, the transcription factor *GLN3*, recently described in *C. glabrata* as the main regulator of nitrogen catabolite repression (NCR) [92], was strongly upregulated in mice. Interestingly, *GLN3* also seems to negatively regulate the ABC transporters *CDR1* and *CDR2*, both involved in fluconazole resistance [93].

Next to nitrogen, carbon is also essential for survival in the host. Since glucose, the preferred carbon source for most yeasts, is rarely present extracellularly, other carbon sources must be used. When carbon sources are not readily available, yeasts use an adapted version of the Krebs cycle for its metabolism, the glyoxylate cycle, which bypasses the CO_2_ expelling steps of the pathway [68]. In our work, we observed a strong upregulation of one enzyme in this cycle, *MLS1*, while the other predicted enzyme, *ICL1* was downregulated in the host. Deletion of *MLS1* led to a significant decrease in virulence in the UTI model. Interestingly, the *in vitro* defect of *MLS1* deletion in *C. glabrata* was not as strong as in *S. cerevisiae*. In baker’s yeast, the deletion mutant cannot grow on acetate as the sole carbon source, independent of the richness of the medium [68]. The growth defect in *C. glabrata* only occurs on minimal medium, not on rich medium. Possibly *C. glabrata* can more efficiently use specific amino acids as a carbon source, as well as a nitrogen source [18]. As mentioned earlier, *ICL1* is downregulated in our study *in vivo* as compared to *in vitro* conditions. In *C. albicans,* this gene was shown to be important for survival inside macrophages [94]. It is either possible that the *ICL1* gene is incorrectly predicted from the genome or that its activity is regulated post-translationally in the host in *C. glabrata*. It would be interesting to further address how *ICL1* deletion affects virulence and how the glyoxylate cycle is regulated in *C. glabrata*.

A fourth gene, *VMA22*, is involved in assembly of the vacuolar ATPase (V-ATPase), which is essential for acidification of this organelle. Acidification of cell organelles creates an electrochemical gradient, which is used, in protein sorting, endocytosis and storage of nutrients [95,96]. V-ATPases are conserved in fungi, plants, and animals, yet some assembly factors are kingdom specific, such as *VMA22* [95]. Deletion of *VMA22* in *S. cerevisiae* results in several growth defects on, among others, glycerol, hydrogen peroxide, alkaline pH, and boric acid [71,97–99]. The *C. albicans* ortholog was unviable in a large-scale survey [100]. Surprisingly, *VMA22* deletion in the present study did not result in any growth defects on the tested media, even though organelle acidification has been linked to oxidative stress resistance and virulence in *C. glabrata* [60,101]. *VMA22* function may differ in *C. glabrata* compared to *S. cerevisiae* and *C. albicans*. Interestingly, in the transposon mutagenesis of Gale *et al*. [102], *VMA22* was scored as a putative essential gene in *C. glabrata*. Further studies are needed on the function of *VMA2*2, given that its deletion resulted in reduced virulence in the UTI model.

Since only a few antifungal targets are known in *C. glabrata*, this work may help the development of potential novel antifungals. Here, we identified four new virulence genes in this species. All four proteins (Dur1,2, Gap1, Mls1, and Vma22) show low identity to human proteins, and as such have potential as antifungal targets. As mentioned earlier, *DUR1,2* was shown to be involved in virulence in both *C. albicans* and *C. glabrata* [85]. Its enzymatic function makes it possible to use existing strategies in development of potential inhibitors. Furthermore, the glyoxylate cycle, of which *MLS1* is a part, does not exist in animals, making it another potentially interesting target.

Our work does confirm that *C. albicans* and *C. glabrata* use divergent different infection strategies, which is reflected by different transcriptional profiles in the host. Recent transcriptional studies performed with different *Candida* spp. incubated with whole blood confirm this observation [103]. *C. albicans* relies heavily on morphogenetic switching, candidalysin secretion and defence against reactive oxygen species (ROS) [30], two of which are absent in *C. glabrata*. The defence against ROS is mainly carried out by superoxide dismutase (SOD) and glutathione biosynthesis and recovery genes in *C. albicans*. Interestingly, *C. albicans* expresses multiple orthologs of both genes, while only one of each is identified in *C. glabrata*. *C. glabrata* does show anti-ROS activity [104], yet neither of the genes involved is upregulated *in vivo* in our work. *C. glabrata* seems to depend mainly on metabolic adaptation to the host environment and expression of adhesins. Both adhesion and metabolic flexibility are also considered as virulence attributes in *C. albicans* [11].

## Conclusions

In conclusion, fungal RNA enrichment and subsequent sequencing is a powerful tool to obtain a full view of the genes and pathways that are differentially regulated during growth inside a host. We show that two classes of virulence genes, the yapsins and the adhesins, probably have a niche-specific expression profile. Furthermore, we confirmed the importance of metabolic flexibility and the ability to use different nitrogen and carbon sources for the virulence capacity of *C. glabrata*. Three out of the four virulence genes identified in this work belong to important, fungal-specific metabolic pathways and therefore may represent useful antifungal targets. Additionally, we illustrate differences between the infection strategy of *C. glabrata* compared to *C. albicans*, and highlight specific virulence genes in either species not having direct homologs in the other.

## Supplementary Material

Supplemental Material

## Data Availability

The authors confirm that the data supporting the findings of this study are available within the article and its supplementary materials.
