# Supplementary material for: Using in vivo transcriptomics and RNA enrichment to identify genes involved in virulence of Candida glabrata
Source: Virulence. 2022 Jul 31;13(1):1285–303. doi: 10.1080/21505594.2022.2095716 (PMC9348041; doi:10.1080/21505594.2022.2095716)
Supplement: Supplemental Material [file KVIR_A_2095716_SM2453.zip › Supplementary/Supplememntary Tables (S1-S5).docx]

**Table S1**: Strains used in this work

| **Strain name** | **Parent strain** | **Genotype** | **Reference** |
| --- | --- | --- | --- |
| SSY21 | DSY562 | p*TEF1*-*Cg*LUC ::*SAT1* | [1] |
| ATCC2001 | NA | NA | [2] |
| G366 | ATCC2001 | *trp1∆*::*TRP1 dur1,2∆*::*NAT1* | [3] |
| G367 | ATCC2001 | *trp1∆*::*TRP1-DUR1,2 dur1,2∆*::*NAT1* | [3] |
| DSY5647 | ATCC2001 | *mls1∆*::*NAT1* | This work |
| SSY61 | ATCC2001 | *mls1∆*::*NAT1 + MLS1*::*HYGB* | This work |
| DSY5652 | ATCC2001 | *gap1∆*::*NAT1* | This work |
| SSY62 | ATCC2001 | *gap1∆*::*NAT1+GAP1*::*HYGB* | This work |
| DSY5661 | ATCC2001 | *vma22∆*::*NAT1* | This work |
| SSY63 | ATCC2001 | *vma22∆*::*NAT1+VMA22*::*HYGB* | This work |

Abbreviations: NA: not available

**Table S2: qPCR primers**

| **Gene** | **Forward** | **Reverse** | **Taqman probe** |
| --- | --- | --- | --- |
| *ATH1* | ATH1-F: CCGAAGGTTGTAATATATAAGTA | ATH1-R: GCCCTCTTATGATTAGAAAG | ATH1-P: TCCGAATACAACAATGACGAGCCTA |
| *DUR1,2* | DUR12-F: GAGGATTTAATGGTCGAG | DUR12-R: GCTAGAACACCAATAGTC | DUR12-P: ATGTCTTGTCTCTTCCTCAGCAGTT |
| *GAP1* | GAP1-F: CAGTCGTACTTGTCATTC | GAP1-R: CCTTGTAGACATCATCTG | GAP1-P: TCCTGGCGTTCTACTTCGGTC |
| *MLS1* | MLS1-F: GCTTGAGAACAGACAAGA | MLS1-R: GACCAGTGATCTCAGTAG | MLS1-P: ATTAGACAGCGGCAACTTCAAGC |
| *VMA22* | VMA22-F: GATCCGATACTTATGTTTGG | VMA22-R: TCAGGAGTTTCACAATTATG | VMA22-P: AACGCCGCAGACACTAAGGA |
| B1J91_K00825g | ScSER2-F: CGTGCCAACTTACTTGAG | ScSER2-R: CCACTAACGTGCATTGATG | ScSER2-P: TCGTTACCACCGTCACCAATCA |
| B1J91_K02035g | ScYER134C-F CAGGATATGTCTATTCGATGA | ScYER134C-R: GGTACAGAGACCACGTAG | ScYER134C-P: TCCGTATGTATATGAACTTGACTCCGT |

**Table S3: Deletion mutant construction and PCR check**

| **Construct** | **fragment 1** | **fragment 2** | **fragment 3** | **fusion** | **check** | |
| --- | --- | --- | --- | --- | --- | --- |
| *GAP1* | GAP1-P1: CAAGATATAGAAGACCCGAGCTG | GAP1-P5: AGAGTACGATCTTAAAAGAAGACATGGAGGCCCAGAATAC | GAP1-P4: TACTAACGCCGCCATCCAGTGAGCCAAAAGACCGGTTTCC | GAP1-P7: GCAAGAGCAGTGATTATCAGTGAC | GAP1-P1 | GAP1-P3 |
|  | GAP1-P2: GTATTCTGGGCCTCCATGTCTTCTTTTAAGATCGTACTCT | GAP1-P6: GGAAACCGGTCTTTTGGCTCACTGGATGGCGGCGTTAGTA | GAP1-P3: ATTGGGCGTTTTTAATATGGAACTTCTCGA | GAP1-P8 CGGCGGGTAGCCTCAACAAT | NAT1_134_R : GAGCCGTAATTTTTGCTTCG | NAT1_743_F : GTGCTGGTCATTTGTGGTTG |
| *MLS1* | CgMLS1-P1: GGGAGTAAGTCGTAACTCTCCT | pDS2020-CgMLS1-F. AAACCAAAATTAACATAAAGATAGCAAACTAAAAAGACATGGAGGCCCAGAATAC | CgMLS1-P3: AATAAATTTATCTTCTCTATCGACTCTATCAT | CgMLS1-P5: GGTTAGAGCGTGTAGGAGAATG | CgMLS1-P1 | CgMLS1-P3 |
|  | CgMLS1-P2: GTATTCTGGGCCTCCATGTCTTTTTAGTTTGCTATCTTTATGTTAATTTTGGTTT | pDS2020-CgMLS1-R. TGTTCACAACTCACAACTCACAACTACTGGATGGCGGCGTTAGTA | CgMLS1-P4: TACTAACGCCGCCATCCAGTAGTTGTGAGTTGTGAGTTGTGAACA | CgMLS1-P6: GTCACGTTGTCTTGTGCGCG | NAT1_134_R | NAT1_743_F |
| *VMA22* | VMA22-P1: TGTCGCCAATTAGTGGATACTTATTATG | VMA22-P5: AAGTAATGAAGGATCATGACGACATGGAGGCCCAGAATAC | VMA22-P4: TACTAACGCCGCCATCCAGTATATCTCGGGCATTTCATGA | VMA22-P7 : TCATGTAAATATATAGCTAGATAGTATCATACAG | VMA22-P1 | VMA22-P3 |
|  | VMA22-P2: GTATTCTGGGCCTCCATGTCGTCATGATCCTTCATTACTT | VMA22-P6: TCATGAAATGCCCGAGATATACTGGATGGCGGCGTTAGTA | VMA22-P3: ATCTGACATATTGTGAAATTATACTAACCTAAC | VMA22-P8 : ATACTTTCTTCTCAGTCCAGGCTTTTG | NAT1_134_R | NAT1_743_F |

**Table S4:** Guides for gene deletions

| Gene | Sequence |
| --- | --- |
| *GAP1* | CACTTGCAGATGATCGCCAT |
| *MLS1* | GCCAAAGATGGAGCATCACT |
| *VMA22* | TATGATCCGATACTTATGTT |

**Table S5: Reintegrant construction**

| **Reintegrant** | **Fwd** | **Rev** |
| --- | --- | --- |
| *GAP1* | pGAP1-Fwd-SacI : AATTCGGAGCTCGTATACAAGATATAGAAGACCC | tGAP1-REV-SpeI : AATTCGACTAGTAAGCCCAGATCTCATTCT |
| *MLS1* | pMLS1-FWD-SacI : AATTCGGAGCTCGACTATTAAACCATCTTAAAGTAGT | tMLS1-REV-SpeI : AATTCGACTAGTCTATCATGTCACGTTGTCT |
| *VMA22* | pVMA22-SacI-FWD : AATTCGGAGCTCCAAGACTTTGAAGTCAAAAGG | tVMA22-SpeI-REV : AATTCGACTAGTATCTGACATATTGTGAAATTATAC |

**References**

1. Schrevens S, Sanglard D. Investigating Candida glabrata Urinary Tract Infections (UTIs) in Mice Using Bioluminescence Imaging. J Fungi (Basel). 2021 Oct 9;7(10).

2. Schwarzmuller T, Ma B, Hiller E, et al. Systematic phenotyping of a large-scale Candida glabrata deletion collection reveals novel antifungal tolerance genes. PLoS Pathog. 2014 Jun;10(6):e1004211.

3. Sprenger M, Brunke S, Hube B, et al. A TRP1-marker-based system for gene complementation, overexpression, reporter gene expression and gene modification in Candida glabrata. FEMS Yeast Res. 2021 Jan 6;20(8).
